# Incidence and survival impact of pulmonary arterial hypertension among patients with systemic lupus erythematosus: a nationwide cohort study

**DOI:** 10.1186/s13075-019-1868-0

**Published:** 2019-03-27

**Authors:** Hung-An Chen, Tsai-Ching Hsu, Su-Ching Yang, Chia-Tse Weng, Chun-Hsin Wu, Chien-Yao Sun, Chun-Yu Lin

**Affiliations:** 1 Division of Allergy-Immunology-Rheumatology, Department of Internal Medicine, Chi Mei Medical Center, Tainan, Taiwan; 2 Chia Nan University of Pharmacy and Science, Tainan, Taiwan; 3 Institute of Biochemistry, Microbiology and Immunology, Chung Shan Medical University, Taichung, Taiwan; 4 Department of Nursing, National Tainan Institute of Nursing, Tainan, Taiwan; 5 Department of Internal Medicine, National Cheng Kung University Hospital, College of Medicine, National Cheng Kung University, No.138, Sheng Li Road, Tainan, 704 Taiwan

**Keywords:** Pulmonary arterial hypertension, Systemic lupus erythematosus, Incidence, Prognosis

## Abstract

**Background:**

No population-based study has investigated the cumulative incidence of pulmonary arterial hypertension (PAH) in patients with newly diagnosed systemic lupus erythematosus (SLE) or the survival impact of PAH in this population.

**Method:**

We used a nationwide database in Taiwan and enrolled incident SLE patients between January 1, 2000, and December 31, 2013. The cumulative incidence of PAH in the SLE patients and the survival of these patients were estimated by the Kaplan-Meier method. Potential predictors of the development of PAH were determined using a Cox proportional hazards regression model.

**Results:**

Of 15,783 SLE patients, 336 (2.13%) developed PAH. The average interval from SLE diagnosis to PAH diagnosis was 3.66 years (standard deviation [SD] 3.36, range 0.1 to 13.0 years). Seventy percent of the patients developed PAH within 5 years after SLE onset. The 3- and 5-year cumulative incidence of PAH were 1.2% and 1.8%, respectively. Systemic hypertension was an independent predictor of PAH occurrence among the SLE patients (adjusted hazard ratio 2.27, 95% confidence interval 1.59–2.97). The 1-, 3-, and 5-year survival rates of SLE patients following the diagnosis of PAH were 87.7%, 76.8%, and 70.1%, respectively.

**Conclusions:**

PAH is a rare complication of SLE and the majority of PAH cases occur within the first 5 years following SLE diagnosis. Systemic hypertension may be a risk factor for PAH development in the SLE population. The overall 5-year survival rate after PAH diagnosis was 70.1%.

## Introduction

Pulmonary arterial hypertension (PAH) is a progressive and life-threatening disease. PAH is defined as an elevated mean pulmonary arterial pressure ≥ 25 mmHg at rest, a pulmonary artery wedge pressure ≤ 15 mmHg, and an increase in pulmonary vascular resistance [1]. A large number of diseases are associated with PAH, and connective tissue disease-associated PAH (CTD-PAH) is the second most common cause of PAH after idiopathic PAH in Western countries [2]. The true incidence and prevalence of PAH in systemic lupus erythematosus (SLE) patients are unknown, but given that a national registry-based study from the UK identified only 35 patients with SLE-PAH [3], PAH is considered to occur in less than 1% of SLE patients [4]. Due to the low incidence of SLE-PAH and the small proportion of CTD-PAH patients affected, the European Respiratory Society does not recommend annual screening for PAH in SLE patients.

In contrast, the distribution of various connective tissue diseases contributing to PAH appears to be different in Asian countries. Recent epidemiological studies from Japan, Korea, and China revealed that SLE is the leading cause of PAH among the spectrum of pulmonary hypertension diseases related to CTDs rather than systemic sclerosis [5–7]. Thus, the characteristics and prognosis of Asian SLE patients with PAH may differ from those of patients in Western countries. However, few population-based studies have evaluated the incidence of PAH among patients with SLE and the long-term survival of these patients in Asia. This prompted us to explore the incidence and the survival impact of PAH in a longitudinal cohort of SLE patients using a national insurance database in Taiwan. We also examined the risk factors associated with PAH development in SLE.

## Method

### Data source

The study was designed as a retrospective cohort study and used data extracted from Taiwan’s National Health Insurance Research Database (NHIRD) (http://nhird.nhri.org.tw/en/index.html). The Taiwan National Health Insurance (NHI) program was initiated on March 1, 1995, and 99% of the Taiwanese population (~ 23 million individuals) are enrolled in the program, making the NHIRD one of the largest and most comprehensive databases in the world.

These databases are maintained by the National Health Research Institute of Taiwan and are provided to scientists for academic research purposes. The NHIRD contains the detailed health care information of each enrollee, including the demographics of beneficiaries, dates of clinical visits, inpatient records, ambulatory care records, and diagnostic codes of interest. The NHIRD has been utilized extensively for epidemiological studies [8, 9]. The Institutional Review Board of National Cheng Kung University Hospital approved the protocol of this study [A-EX-104-045]. Informed consent was not required because the datasets are devoid of personally identifiable information.

### Participants

The International Classification of Diseases, ninth revision, clinical modification (ICD-9-CM code) was used to identify diseases and comorbid conditions in our study. Patients in the study were defined as having SLE only if they received diagnosis code 710.0 and had a catastrophic illness certificate. In Taiwan, patients with SLE can apply for catastrophic illness certificates from the Bureau of National Health Insurance to be exempted from co-payments when seeking health care related to SLE. The certificate can be issued to SLE patients only when their medical records, laboratory data, and imaging results have been reviewed by certified rheumatologists and confirmed to fulfill the 1997 American College of Rheumatology revised criteria for classification of systemic lupus erythematosus. Only including patients with catastrophic illness certificates in the study ensures that the diagnosis of SLE was valid.

The SLE patients were selected between January 1, 2000, and December 31, 2013. The index date was the date of the first diagnosis of SLE.

### Comorbidity and outcome measures

Patient characteristics such as age, sex, and comorbidities were retrieved.

Participants were divided into three groups by their age at SLE diagnosis: < 45, 45–64, and ≥ 65. The selected baseline comorbidities included diabetes (ICD-9-CM codes 250.xx), hyperlipidemia (ICD-9-CM codes 270.1–270.4), hypertension (ICD-9-CM codes 401.xx–405.xx), and chronic kidney disease (ICD-9-CM codes 403.xx, 404.xx, 582.xx, 585.xx, 586.xx, 587.xx, and 588.xx). These comorbid medical conditions were identified based on one inpatient claim or three ambulatory claims prior to the date of SLE diagnosis.

Pulmonary arterial hypertension was identified based on the ICD-9-CM code 416.0, which was required to be recorded at least three times in outpatient visits or at least one time in an inpatient care visit. The accuracy of ICD code 416.0 for a diagnosis of PAH was validated with a specificity of 96.7% in a previous study [10]. The code was therefore considered to be credible for the diagnosis of PAH based on the clinical symptoms and results of an echocardiography and/or right heart catheterization. The first date a patient received a diagnosis of PAH was defined as the day of PAH development. Patients with PAH before the diagnosis of SLE were excluded from this study. We also excluded patients with congestive heart failure from the analysis. All patients were followed up until death or the end of the study (December 31, 2013), whichever came first.

### Statistical analysis

Continuous variables are expressed as the mean and standard deviation (SD). Categorical variables are expressed as a number and percentage. The independent Student *t* test or Pearson chi-square test was used to determine differences in continuous and categorical variables, respectively, including demographic data and patient comorbidities, between the SLE patients with PAH and those without PAH. The Kaplan-Meier method was adopted to estimate the cumulative incidence of PAH among the patients with SLE. Survival rates were also calculated using the Kaplan-Meier method. The log-rank test was used to determine differences between survival curves. Cox proportional hazard regression was used to identify the predictors of PAH development and mortality among the SLE patients, and these results were reported as hazard ratios (HRs) with 95% confidence intervals. Multivariate analyses were performed adjusting for age, sex, and selected comorbidities. A two-sided *P* value of 0.05 was considered significant. All data processing and statistical analyses were performed in Stata 13 software (StataCorp, College Station, TX, USA).

## Results

### Patients’ characteristics

**Fig. 1:**
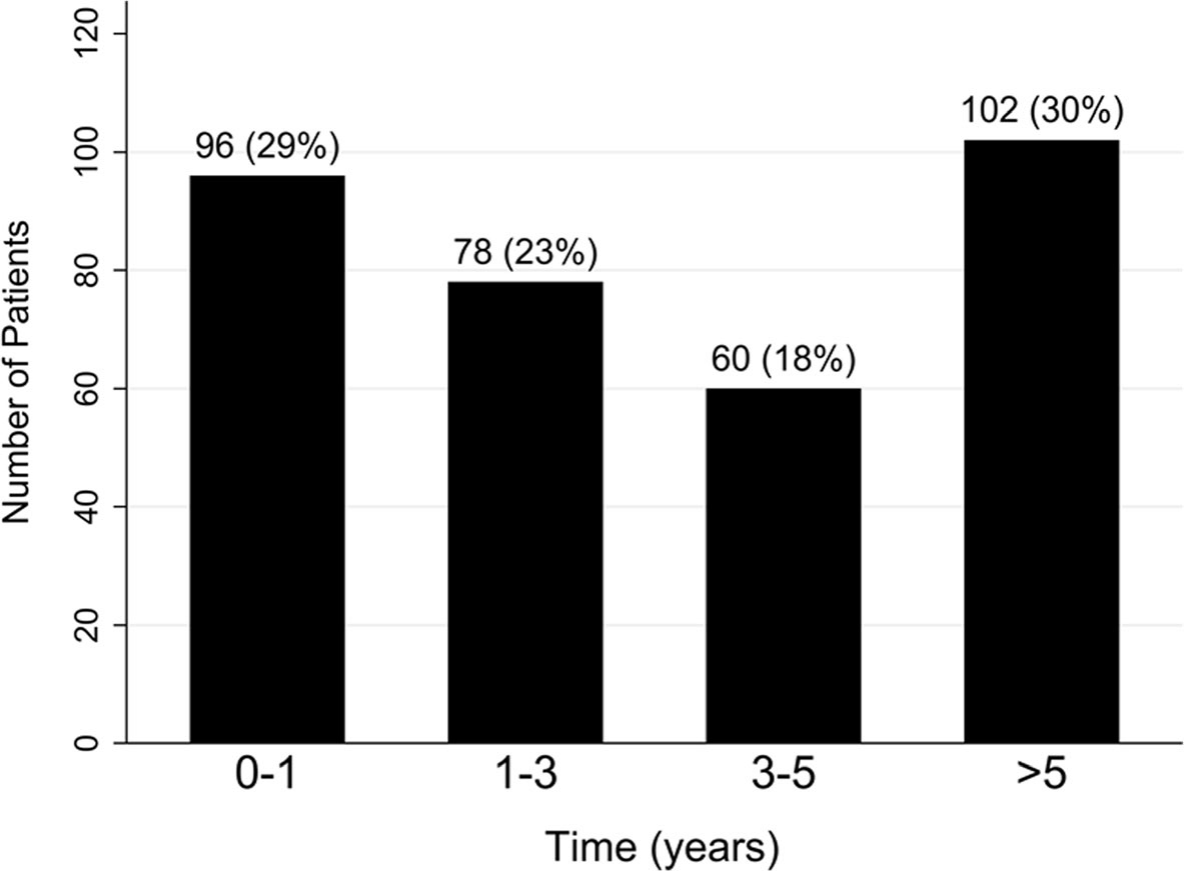
Number of patients with different intervals from systemic lupus erythematosus diagnosis to pulmonary arterial hypertension diagnosis

A total of 15,783 patients with SLE were identified during the study period, 336 of whom (2.13%) developed PAH. Female patients accounted for approximately 90% of both groups (Table 1). No significant differences were found between the two groups in terms of age at SLE diagnosis. The interval from SLE diagnosis to PAH diagnosis ranged from 0.1 to 13.0 years. The mean and median duration from SLE diagnosis to PAH diagnosis was 3.66 years (SD 3.36) and 2.81 years (interquartile range 0.69–5.79), respectively. We stratified the patients into four groups by the interval from SLE diagnosis to PAH diagnosis and calculated the percentage for each group (Fig. 1). The majority (70%) of patients developed PAH within 5 years after the diagnosis of SLE. Comorbidities were relatively low in both groups. Hypertension was significantly higher among the patients with both SLE and PAH.

**Table 1:** Demographic information and characteristics of systemic lupus erythematosus (SLE) patients with and without pulmonary arterial hypertension (PAH)

Characteristic	SLE with PAH (*n* = 336)	SLE without PAH (*n* = 15,447)	*P* value
Gender			0.01
Female	309 (91.9)	13,474 (87.2)	
Male	27 (8.04)	1973 (12.77)	
Age at diagnosis of SLE, years	36.81 ± 16.76	37.08 ± 16.99	0.77
Age groups at diagnosis of SLE, years			0.45
< 45	246 (73.21)	10,842 (70.19)	
45–64	65 (19.35)	3409 (22.07)	
≥ 65	25 (7.44)	1196 (7.74)	
Age at diagnosis of PAH, years	40.47 ± 16.60	–	
Interval between SLE and PAH, years	3.66 ± 3.26	–	
Mean follow-up duration, years (min-max)	7.4 (0.1–13.9)	6.5 (0.1–13.9)	< 0.001
Comorbidity			
Diabetes mellitus	15 (4.46)	774 (5.01)	0.64
Hypertension	72 (21.43)	2456 (15.90)	0.006
Dyslipidemia	21 (6.25)	1171 (7.58)	0.36
Chronic kidney disease	22 (6.55)	1357 (8.78)	0.15

Data are presented as *n* (%) or mean ± SD unless otherwise noted

### Incidence of and risk factors for PAH in SLE patients

**Fig. 2:**
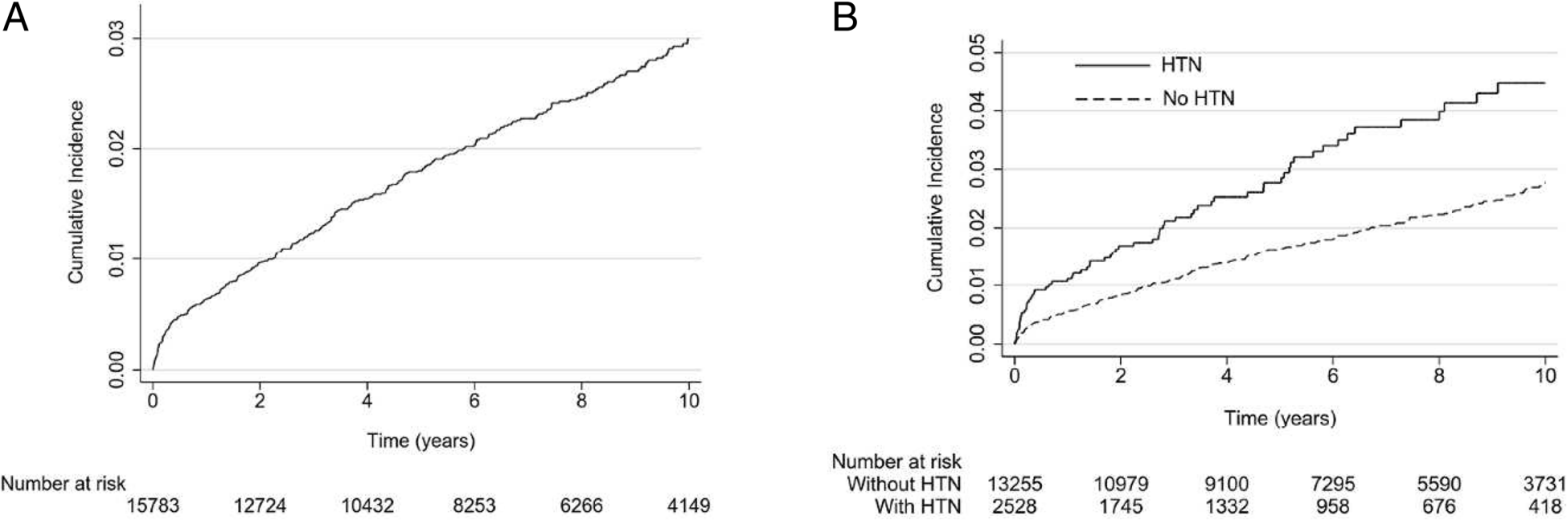
Cumulative incidence of pulmonary arterial hypertension in patients with new diagnosis of systemic lupus erythematosus. **a** Whole cohort. **b** Comparison of patients based on the presence of systemic hypertension (HTN)

The cumulative incidence of PAH among SLE patients is illustrated in Fig. 2a. The 3- and 5-year incidence of PAH were 1.2% and 1.8%, respectively. Figure 2b shows that the SLE patients with hypertension had a significantly higher risk of developing PAH than those without hypertension (log-rank test, *p* <  0.001). Table 2 presents the risk factors for the occurrence of PAH in the SLE cohorts. Hypertension was still a predictor of PAH after adjusting for age, sex, and other comorbidities (adjusted HR 2.17, 95% CI 1.59–2.97). Another independent risk factor for PAH was female gender (adjusted HR 1.56, 95% CI 1.05–2.32). Diabetes was not a risk factor for PAH occurrence among the SLE patients (adjusted HR 0.99, 95% CI 0.56–1.72).

**Table 2:** Crude and adjusted hazard ratios of predictors of pulmonary arterial hypertension (PAH) in systemic lupus erythematosus (SLE) patients

Variables	Crude HR (95% CI)	*P* value	Adjusted HR (95% CI)	*P* value
Sex				
Female	1.51 (1.01–2.23)	0.04	1.56 (1.05–2.32)	0.03
Male	1 (reference)		1 (reference)	
Age group, years				
< 45	1 (reference)		1 (reference)	
45–64	0.94 (0.72–1.24)	0.7	0.80 (0.60–1.08)	0.15
≥ 65	1.45 (0.96–2.19)	0.07	1.05 (0.66–1.68)	0.82
Comorbidity				
Diabetes mellitus	1.21 (0.72–2.04)	0.46	0.99 (0.56–1.72)	0.97
Hypertension	1.80 (1.39–2.34)	< 0.001	2.17 (1.59–2.97)	< 0.001
Dyslipidemia	1.00 (0.64–1.55)	0.99	0.80 (0.49–1.28)	0.35
Chronic kidney disease	0.81 (0.52–1.25)	0.34	0.66 (0.42–1.04)	0.07

Adjusted for age, sex, and the listed comorbidities

*HR* hazard ratio

### Survival of SLE patients following PAH diagnosis

**Fig. 3:**
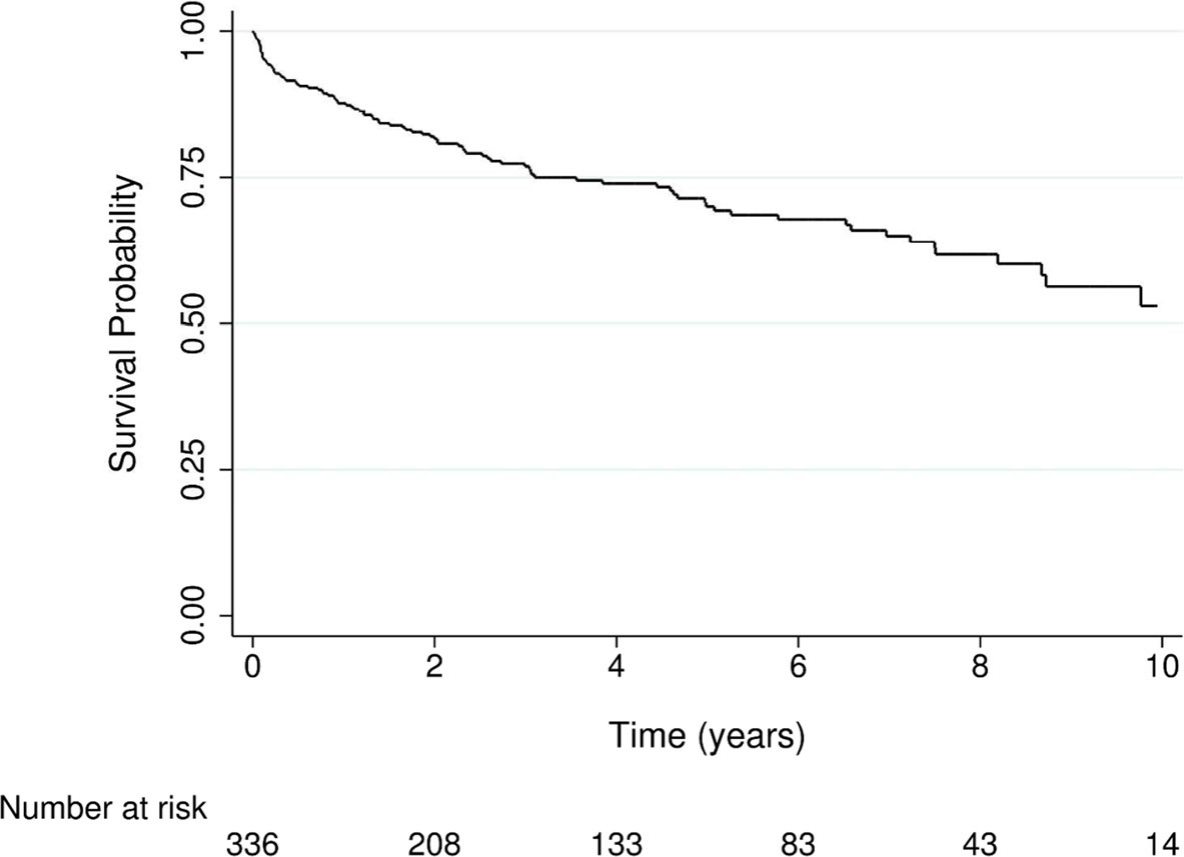
Survival probability of patients with systemic lupus erythematosus after the diagnosis of pulmonary arterial hypertension

The 1-, 3-, and 5-year survival rates of SLE patients after the diagnosis of PAH were 87.7%, 76.8%, and 70.1%, respectively (Fig. 3). PAH was significantly associated with higher mortality among SLE patients (Table 3). A multivariate Cox model showed that the mortality risk among the patients with PAH increased 120% compared to that among those without PAH (adjusted HR 2.20, 95% CI 1.78–1.71) (Table 3). Other independent predictors of death in SLE patients included male gender, old age, diabetes, hypertension, and chronic disease.

**Table 3:** Crude and adjusted hazard ratios of predictors for mortality among systemic lupus erythematosus (SLE) patients

Variables	Crude HR (95% CI)	*P* value	Adjusted HR (95% CI)	*P* value
Pulmonary arterial hypertension	2.09 (1.09–1.57)	< 0.001	2.20 (1.78–1.71)	< 0.001
Sex				
Female	1 (reference)		1 (reference)	
Male	1.83 (1.64–2.05)	< 0.001	1.29 (1.15–1.44)	< 0.001
Age group, years				
< 45	1 (reference)		1 (reference)	
45–64	2.28 (2.04–2.54)	< 0.001	1.88 (1.68–2.11)	< 0.001
≥ 65	9.42 (8.46–10.09)	< 0.001	6.07 (5.32–6.92)	< 0.001
Comorbidity				
Diabetes mellitus	4.27 (3.76–4.85)	< 0.001	1.59 (1.38–1.83)	< 0.001
Hypertension	3.82 (3.48–4.19)	< 0.001	1.48 (1.32–1.67)	< 0.001
Dyslipidemia	2.24 (1.96–2.55)	< 0.001	0.85 (0.74–0.98)	0.033
Chronic kidney disease	2.99 (2.68–3.34)	< 0.001	1.88 (1.67–2.72)	< 0.001

Adjusted for age, sex, and the listed comorbidities

*HR* hazard ratio

## Discussion

To the best of our knowledge, this is the first nationwide, population-based, long-term follow-up study to explore the incidence of PAH among patients with SLE as well as the survival impact of PAH. We found that the 5-year incidence of PAH was 1.8%. Approximately 70% of PAH cases occurred within 5 years after the diagnosis of SLE. Another interesting finding was that systemic hypertension predicted the development of PAH among SLE patients. The survival rates at 3 and 5 years after the diagnosis of PAH were 76.8% and 70.1%, respectively. Therefore, PAH confers a higher mortality risk on SLE patients.

The reported prevalence of PAH in SLE patients ranges from 0.5 to 14% [11–16]. However, most of the previous studies investigating the incidence and prevalence of SLE-PAH were registry-based or used data from tertiary referral centers, and the numbers of SLE-PAH patients in these studies were usually small. Referral bias was likely to occur in these studies. Overestimation of the true incidence of PAH is likely in a tertiary hospital-based cohort [11, 13]. In the present study, we utilized a nationwide research database, enrolled 15,783 SLE patients to estimate the cumulative incidence of PAH, and found a 5-year PAH incidence rate of 1.8%. Because our cohort could be representative of the general SLE population, we think our study better reflects the incidence rate than previous studies in Asian countries.

PAH can develop concurrently with the diagnosis of SLE or many years after SLE onset. Occasionally, PAH may be the presenting feature of SLE [17]. In a study using the French pulmonary hypertension registry, half of the patients developed PAH within 5 years after the diagnosis of SLE [18]. The percentage of patients with an interval between their SLE and PAH diagnoses shorter than 5 years in our cohort was even higher at 70%. Our data suggest that clinicians should monitor minor symptoms associated with PAH more closely and arrange appropriate tests for screening in the first 5 years following SLE diagnosis.

Systemic hypertension is not a recognized risk factor for idiopathic pulmonary arterial hypertension [1]. However, we found that systemic hypertension can be an independent predictor of the future development of PAH in the SLE population after adjusting for sex, age, and other common comorbidities in a multivariable model. The pathogenesis of systemic hypertension in SLE is complex and not fully understood. A combination of traditional (e.g., age, body mass index, and smoking) and disease-specific factors (immune system dysregulation, inflammatory cytokines, and drug side effects) may contribute to systemic hypertension in SLE patients [19–21]. Moreover, SLE-associated PAH also has an immune/inflammatory component. Vasculitis or capillaritis of the pulmonary vessels has been shown to lead to vascular remodeling, damage, and subsequent elevated pulmonary artery pressure [22, 23]. Patient responsiveness to immunosuppressive agents also suggests a role of inflammation in the pathogenesis of PAH in SLE patients [24]. Thus, immune dysregulation and inflammatory cytokines may link systemic hypertension and pulmonary hypertension in patients with SLE. Another possibility for the association between systemic hypertension and PAH in our SLE cohort is that systemic hypertension would result in the development of congestive heart failure and subsequent pulmonary hypertension due to left heart disease (group II pulmonary hypertension). However, this possibility is low in our analysis as pulmonary hypertension due to left heart disease would be coded as ICD-9-CM 416.8 and we only used ICD-9-CM 416.0 to identify patients with PAH. Moreover, diabetes is associated with congestive heart failure [25] and may contribute to the development of pulmonary hypertension due to left heart disease, but there was no association between diabetes and PAH among the SLE patients in our multivariable model. We believe that our observation of the linkage between systemic hypertension and PAH among the patients with SLE could not be attributed to confounding by congestive heart failure. Our findings imply that physicians should have increased awareness of PAH in SLE patients accompanied by systemic hypertension.

The prognosis of SLE patients with PAH differs between Western and Asian countries. In a recently published registry study from France, the 3- and 5-year overall survival rates after PAH diagnosis were 89.4% and 83.9%, respectively [18]. A UK SLE-PAH cohort study reported a similar result, with an 85% survival rate at 5 years [18, 26]. A Chinese study of 111 SLE-PAH patients described 3- and 5-year survival rates of 81.3% and 61.0%, respectively [27]. Another Chinese study, which enrolled 310 SLE-PAH patients from 14 referral centers, revealed 3- and 5- year survival rates of 84.8% and 72.9%, respectively [28]. The result of 5-year survival rate from the multicenter study was quite similar to our study. A single-center study conducted in Korea reported 3- and 5-year survival rates of 79.0% and 60.2%, respectively [29]. Our cohort of 336 SLE-PAH patients also indicated that these patients had a worse prognosis than the SLE patients in Western countries. The reasons for the difference in survival between Western and Asian patients require further investigation.

A strength of our study is that we used a nationwide database that included medical records for 99% of the residents of Taiwan. Thus, we can estimate the cumulative incidence of PAH among SLE patients rather than the prevalence. The main limitation of this study was the lack of important biomarkers for SLE, such as complement level and antibody status. The disease activity of SLE could not be evaluated in our study. Hemodynamic measurements for PAH were also not available in our database. Another limitation worth mentioning is that our data source is based on a claims database, and the identification of PAH is dependent on the ICD-9-CM codes. Misclassification bias cannot be completely excluded. This bias is an inherent limitation of administrative database studies. Nevertheless, the accuracy of ICD-9-CM code 416.0 for PAH has been validated in this nationwide database in Taiwan and has a specificity of 96.7% [10], although residual misclassification bias may still exist as not all of the patients in our study received right heart catheterization. Therefore, we think our results are robust against coding errors.

## Conclusion

PAH is a rare complication of SLE and has a 5-year cumulative incidence of 1.8%. Seventy percent of PAH cases occur within the first 5 years after SLE onset. The development of PAH in SLE patients is a poor prognostic factor, but systemic hypertension may be a predictive factor for PAH in the SLE population. The overall survival rate for an SLE patient with PAH is worse in Asian countries than in Western countries.

## Abbreviations

CTD: Connective tissue disease
HR: Hazard ratio
HTN: Hypertension
ICD-9-CM: International Classification of Diseases, ninth revision, clinical modification
NHI: National Health Insurance
NHIRD: National Health Insurance Research Database
PAH: Pulmonary arterial hypertension
SLE: Systemic lupus erythematosus
